# Subgenera of *Charidotella* Weise with description of a new subgenus and species from Brazil (Coleoptera, Chrysomelidae, Cassidinae, Cassidini)

**DOI:** 10.3897/zookeys.506.8770

**Published:** 2015-05-28

**Authors:** Lukáš Sekerka, Lech Borowiec

**Affiliations:** 1 Department of Entomology, National Museum, Cirkusová 1740, CZ-193 00 Praha 9, Czech Republic; 2 Department of Zoology, Faculty of Science, University of South Bohemia, Branišovská 31, CZ-370 05 České Budějovice, Czech Republic; 3 Department of Biodiversity and Evolutionary Taxonomy, University of Wrocław, Przybyszewskiego 63/77, 51-148 Wrocław, Poland

**Keywords:** Entomology, taxonomy, new subgenus, new species, new combination, Neotropical Region, Brazil

## Abstract

A new subgenus and species, Charidotella (Chapadacassis subgen. n.) paradoxa **sp. n.** is described and figured from the Chapada plateau in Mato Grosso, Brazil. Subgenera of *Charidotella* Weise, 1896 are listed, supplemented with basic data, diagnostic table, and a key is proposed. Based on a study of respective type material following new combinations are proposed: Charidotella (Philaspis) stulta (Boheman, 1855), **comb. n.**, Charidotella (Xenocassis) amoenula (Boheman, 1855), **comb. n.**, Charidotella (Xenocassis) cyclographa (Boheman, 1855), **comb. n.**, Charidotella (Xenocassis) discoidalis (Boheman, 1855), **comb. n.**, Charidotella (Xenocassis) incerta (Boheman, 1855), **comb. n.**, Charidotella (Xenocassis) purpurea (Linnaeus, 1758), **comb. n.**, Charidotella (Xenocassis) myops (Boheman, 1855), **comb. n.** (all previously placed in the nominotypical subgenus), and *Plagiometriona cingulata* (Boheman, 1862), **comb. n.** (from Charidotella (Xenocassis)).

## Introduction

New World Cassidini comprises currently 726 species in 46 genera. Brazil is the country with richest fauna represented by 402 species, 252 of them so far known only from Brazil (Borowiec and Świętojańska 2015). Most likely the number of endemic taxa will be much lower as many species occur also in neighbouring countries. Particularly Bolivia and Venezuela are quite poorly explored regarding Cassidinae fauna and our recent research indicates that many species so far known only from Brazil are present in these countries too (Windsor and Sekerka, unpubl. data).

Dry regions of Southern America are poorly collected and many species are known only from small areas while their range is in fact large. This is particularly true for western Bolivia and central-west Brazil (Windsor and Sekerka, unpubl. data). The Chapada plateau in Mato Grosso is perhaps one of the most interesting areas in this part of Southern America and probably hides numerous undescribed taxa. Several cassidines were described recently by Świętojańska and Borowiec (1995, 1996, 1999) and Borowiec (2004). In the material studied recently we found a new species belonging to an undescribed peculiar subgenus of *Charidotella* Weise, 1896 characterized by completely irregular punctation of the elytra, a very rare morphological feature in New World Cassidini.

Weise (1896) proposed *Charidotella* for a single species, *Charidotella zona* (Fabricius, 1801), while he also created *Metriona* Weise, 1896 where he placed most species currently classified in *Charidotella*. Spaeth (1914) downgraded *Charidotella* to subgenus of *Metriona* and designated *Metriona elatior* (Klug, 1829) as the type species of the latter. He also included six more species in *Charidotella*, all having pattern on the ventral side of the elytral disc. Spaeth (1942) raised *Charidotella* to genus rank and listed eleven species in it. Meantime, Spaeth described several genera (*Philaspis* Spaeth, 1913, *Xenocassis* Spaeth, 1936 and *Metrionaspis* Spaeth, 1942) for species previously classified in *Coptocycla* Chevrolat, 1836 or *Metriona*. Subsequently Hincks (1952) placed them as subgenera of *Charidotella* and validated one more subgenus *Charerocassis* Spaeth in Hincks, 1952 following Spaeth’s unpublished manuscript for Wytsman’s Genera Insectorum. Borowiec (1989) placed *Metrionaspis* as subgenus of *Charidotella*, proposed a key to the subgenera and the first catalogue of the genus. Most recently, Windsor et al. (1992) considered *Xenocassis* as a separate genus, however, this change was not accepted and *Xenocassis* remained as subgenus of *Charidotella* (e.g. Borowiec 1999).

Currently *Charidotella* comprises 100 species divided in five subgenera (Borowiec and Świętojańska 2015). Identification of subgenera was established mainly on the basis of structure of tarsal claws (simple vs. appendiculate) by Spaeth (1936) and followed by Borowiec (1989). General body shape, convexity of the elytra, and punctation provide good characters too, however, in many cases they are hard to describe to be clearly and easily understood. The structure of tarsal claws proved as yet not fully understood and at least some species have intraspecific variability in presence or absence of the basal tooth on respective claw (e.g. Riley 1982, 1986). Besides the key we provide also a diagnostic table (Table 1) to help to recognize subgenera of *Charidotella*.

**Table 1.** Summarizing most important morphological differences among subgenera of *Charidotella* Weise, 1896. Numbers in parentheses indicate number of species possessing particular character when variable within one subgenus.

subgenus character	*Chapadacassis* subgen. n.	*Philaspis* Spaeth, 1913	*Metrionaspis* Spaeth, 1942	*Chaerocassis* Spaeth in Hincks, 1952	*Xenocassis* Spaeth, 1936	*Charidotella* s. str. Weise, 1896
body shape	oval and parallelsided	oval and parallelsided	subtriangular to subcircular	subcircular (3) or oval (3)	circular	variable, mostl subcircular to subtriangular
convexity of elytra	irregular and subgibose	regular and weak	irregular with postscutellar hump	regular and weak	regular and moderate	from regular and low to tuberculate
punctation of elytra	irregular	regular	regular	regular	regular	regular
explanate margin of elytra	densely punctate	sparsely punctate (4) or impunctate (6)	sparsely punctate in humeral area	impunctate (4) or punctate in humeral area (2)	impunctate	impunctate
pattern on underside of elytra	absent	present (6) or absent (4)	absent	absent	absent	present (at least in 32 species) or absent
pattern on disc of elytra	absent	absent	disc uniformly red	disc uniformly red or yellow	usually with ring, rarely yellow	usually yellow
spots on explanate margin of elytra	absent	absent	present	present (5) or absent (1)	absent	absent (61) or present (7)
sides of pronotum	rounded	subangulate	rounded	rounded	rounded	rounded (66) or subangulate (1)
size of eye	large, occupying almost whole side	large, occupying almost whole side	large, occupying almost whole side	large, occupying almost whole side	moderately large occupying 2/3 of side, gena well visible	large, occupying almost whole side, gena sometimes visible but very narrow
antennae	5 basal slim shiny antennomeres + 6 dull and broad	6 basal slim shiny antennomeres + 5 dull and broad	5 basal slim shiny antennomeres + 6 dull and broad	6 basal slim shiny antennomeres + 5 dull and broad	6 basal slim shiny antennomeres + 5 dull and broad	6 basal slim shiny antennomeres + 5 dull and broad
antennomeres II–IV	II and III subequal, IV approx. 1/4 longer than either II and III	II distinctly shorter than III, III and IV subequal	II and III subequal, IV as long as II and III combined	III longer than II and IV longer than III	variable	variable
proclaws	both with small basal tooth	both with large basal tooth	both with large basal tooth	both with large basal tooth	both with small to large basal tooth	both with moderate to large basal tooth
mesoclaws	outer simple, inner with small tooth	outer simple (♂) or with small tooth (♀), inner with large tooth	both simple (♀) or outer with large basal tooth (♂)	♂: both or only inner simple; ♀: all with large tooth or one simple	inner with small to large basal tooth, outer simple or both simple	outer simple (♂) or both with small to large tooth (♀)
metaclaws	both with small basal tooth	both with large basal tooth	inner simple, outer with large basal tooth	♂: inner simple; ♀: both with large tooth or one simple	inner with small to large basal tooth, outer simple or both simple or both with large tooth	both with moderate to large basal tooth

The genus *Charidotella* can be characterized by at least some tarsal claws with a basal tooth, venter of the pronotum without antennal grooves, the clypeus flat or impressed and without distinct grooves, and a broad prosternal process with the apex not strongly expanded laterally. *Charidotella* species are mostly associated with the plant family Convolvulaceae, mainly with the diverse genus *Ipomoea*, however at least one species is associated with Asteraceae (Windsor and Sekerka, unpubl. data).

Label data from the type specimens are cited as they appeared on the labels. Individual labels are separated by a double vertical bar “||” and rows within the label by a single vertical bar “|”.

## Overview of subgenera of *Charidotella*

### Charidotella (s. str.)

Taxon classificationAnimaliaColeopteraChrysomelidae

Weise, 1896

Figs 8–9

Charidotella Weise, 1896: 13.

#### Type species.

*Cassida zona* Fabricius, 1801 by monotypy.

#### Number of species.

67 (Borowiec and Świętojańska 2015, present paper).

#### Key to species.

Borowiec (2007) proposed a key covering 23 species with pattern on the ventral part of the elytral disc.

#### Range.

Canada to Argentina.

#### Distinguishing characters.

Species of the nominotypical subgenus can be separated by all tarsal claws with a basal tooth of variable size, or in males the outer claw of mesotarsi is with small tooth or simple. They also have subcircular to subtriangular body and are more convex in comparison to most other subgenera except *Metrionaspis* and *Chapadacassis* subgen. n. Otherwise the nominotypical subgenus is polymorphic displaying greater variability and some species externally reminds other subgenera. Most species are yellow with or without pattern on the ventral side of the elytral disc which can be variable. After revising most species of *Charidotella* there is no species in the nominotypical subgenus with dark annulus on the upper side of the elytra and all such coloured species are here transferred to *Xenocassis*.

### Chaerocassis

Taxon classificationAnimaliaColeopteraChrysomelidae

Spaeth in Hincks, 1952

Figs 10–11

Charidotella subgen. Chaerocassis Spaeth in Hincks, 1952: 350.

#### Type species.

*Coptocycla marculenta* Boheman, 1855 by original designation.

#### Number of species.

6 (Borowiec and Świętojańska 2015).

#### Key to species.

Not yet proposed.

#### Range.

USA to Panama.

#### Distinguishing characters.

*Chaerocassis* species have subcircular or oval body outline, regularly convex elytra, the base of the elytra distinctly wider than the pronotum and humeral angles moderately projecting anterad. Four species have explanate margin of the elytra with basal and posterolateral spots. One species has outer margin of the elytra black and the type species is uniformly yellow. Males have the outer claw of meso- and metatarsi, or both claws of meso- and the outer claw of metatarsi simple. Females have all claws appendiculate or one of the meso- and metatarsi simple. They are externally close to the nominotypical subgenus but can be easily separated by one of the metaclaws simple and elytra always without pattern on uderside.

### Metrionaspis

Taxon classificationAnimaliaColeopteraChrysomelidae

Spaeth, 1942

Figs 16–17

Metrionaspis Spaeth, 1942: 39; Borowiec 1989: 204 (as subgenus of *Charidotella*).

#### Type species.

*Aspidomorpha rubicunda* Guérin-Méneville, 1844 by monotypy.

#### Number of species.

2 (Borowiec and Świętojańska 2015).

#### Key to species.

Not yet proposed.

#### Range.

*Charidotella rubicunda* is widely distributed through South America from Colombia to Argentina while *Charidotella santaremi* Borowiec, 1995 is so far known only from the state of Pará in Brazil.

#### Distinguishing characters.

The two *Metrionaspis* species have a broadly oval to subtriangular body outline, base of the elytra distinctly wider than pronotum with humeral angles projecting anterad, explanate margin of the elytra with humeral and posterolateral spots, and the elytra with a postscutellar tubercle. Externally both species are very similar to two *Charidotella* s. str. species, *Charidotella tuberculata* (Fabricius, 1775) and *Charidotella ventricosa* (Boheman, 1855), but they can be separated by an impunctate explanate margin of the elytra and claws of the metatarsi in both sexes with a basal tooth. While *Metrionaspis* species have humeral area of the explanate margin punctate and the inner claw of the metatarsi simple in both sexes.

### Philaspis

Taxon classificationAnimaliaColeopteraChrysomelidae

Spaeth, 1913

Figs 14–15

Philaspis Spaeth, 1913: 142; Hincks 1952: 342 (as subgenus of *Charidotella*).

#### Type species.

*Odontionycha seriatopunctata* Spaeth, 1901 designated by Hincks (1952).

#### Number of species.

10 (Borowiec 2004, present paper).

#### Key to species.

Spaeth (1936) covered eight species, Borowiec (2004) covered nine species.

#### Range.

One species in Mexico and Costa Rica, remaining in the southern part of South America.

#### Distinguishing characters.

*Philaspis* species are at first glance easily distinguished by the parallel-sided elytra in combination with subangulate sides of the pronotum. All species are uniformly yellow or have a small black spot in the middle of each elytron.

#### Remarks.

*Charidotella stulta* (Boheman, 1855) was previously classified in the nominotypical subgenus. We recently examined its holotype, preserved in Museum für Naturkunde, Berlin, and found that it belongs to the subgenus *Philaspis* near Charidotella (Philaspis) inculta (Boheman, 1855).

### Xenocassis

Taxon classificationAnimaliaColeopteraChrysomelidae

Spaeth, 1936

Figs 12–13

Xenocassis Spaeth, 1936: 260; Hincks 1952: 342 (as subgenus of *Charidotella*).

#### Type species.

*Coptocycla amoena* Boheman, 1855 by original designation.

#### Number of species.

15 (present paper).

#### Key to species.

Not yet proposed.

#### Range.

Mexico to Peru with most species in the Central America.

#### Distinguishing characters.

*Xenocassis* species can be easily separated from other subgenera by the small eyes covering only 2/3 of lateral sides of the head thus gena is well visible while all other subgenera have large eyes. In addition *Xenocassis* has nearly regularly circular body outline, weakly convex elytra with coarser punctation on lateral slope, and dorsum with ring pattern on the upper side. In extreme cases the ring can form a large discal spot or can be completely vanished thus whole dorsum is uniformly yellow.

#### Remarks.

So far *Xenocassis* was separated from other genera on the basis of the tarsal claws and general body shape. Windsor et al. (1992) were the first who noticed that all species have also small eyes in comparison to other *Chardotella* species. As a result they raised *Xenocassis* to genus in the provided key but unfortunately made no additional comments and their change was not accepted later (e.g. Borowiec 1999). We agree with them that the small size of the eye is diagnostic for *Xenocassis* and found that five species currently classified in the nominotypical subgenus should be transferred to *Xenocassis* based on this character. In addition we found that *Xenocassis* species are very variable regarding the size and presence of tarsal appendages. The genus was based by Spaeth (1936) on the outer claws of the metatarsi simple in both sexes, however, examination of extensive material revealed that even the type species, *Charidotella amoena*, could have the outer claws of the metatarsi with a large basal tooth. Similar situation was found in two other species we had extensive material to study – Charidotella (Xenocassis) ambita (Champion, 1894) and Charidotella (Xenocassis) puella (Boheman, 1855). In both the basal teeth showed variable size even within one population. While the size of the eye is constant. Some species of other subgenera have slightly smaller eyes than others thus they have gena visible but always very narrow while species of *Xenocassis* have gena covering approximately basal third of lateral side of the head.

We consider *Xenocassis* as subgenus of *Charidotella* as the size of the eye is found variable also in some other new world Cassidini genera, e.g. *Charidotis* Boheman, 1855 and *Plagiometriona* Spaeth, 1899.

Last catalogue, Borowiec (1999) listed 10 species in the subgenus *Xenocassis*. We have recently examined types of all species and found that one was wrongly assigned to *Xenocassis*. *Coptocycla cingulata* Boheman, 1862 (type seen in the Natural History Museum, London) was unknown to most authors and have been tentatively placed in *Charidotella* based on the original description (Boheman 1862) and notes published by Champion (1894) in the Cassidinae volume of the Biologia Centrali Americana (Borowiec 1989). It posses all characters of the genus *Plagiometriona* and is here transferred to it as *Plagiometriona cingulata* (Boheman, 1862), comb. n.

During examination of species placed in the nominotypical subgenus we found four which had small eyes and are here transferred to *Xenocassis*: Charidotella (Xenocassis) discoidalis (Boheman, 1855), comb. n., Charidotella (Xenocassis) incerta (Boheman, 1855), comb. n., Charidotella (Xenocassis) purpurea (Linnaeus, 1758), comb. n., and Charidotella (Xenocassis) myops (Boheman, 1855), comb. n. Types of all, with exception of *Charidotella purpurea*, were examined and are preserved in the Naturhistoriska Riksmuseet, Stockholm, Sweden. In addition Boheman (1855) described two more species in the same groups as abovementioned ones and we have strong feeling that they belong to *Xenocassis* too: Charidotella (Xenocassis) amoenula (Boheman, 1855), comb. n. and Charidotella (Xenocassis) cyclographa (Boheman, 1855), comb. n. Unfortunately, we were not able to locate their type specimens thus the transfer is tentative, based on primary descriptions according to which the species should have the circular body shape, the annulus on upper side of the elytra, and coarser punctation on the lateral slope of elytral disc like other *Xenocassis* species.

### Chapadacassis subgen. n.

Taxon classificationAnimaliaColeopteraChrysomelidae

http://zoobank.org/2BC3A84F-44A2-48C0-A888-14EB4101B789

Figs 1–7

#### Type species.

Charidotella (Chapadacassis) paradoxa sp. n. here designated.

#### Etymology.

The genus name is a combination of its type locality, the Chapada plateau and the genus name *Cassida*, gender feminine.

#### Diagnosis.

*Chapadacassis* subgen. n. is well characterized by completely irregular punctation of the elytra, only apical two thirds of sutural row appear more or less regular, while all other *Charidotella* species have mostly regular punctation of the elytra. Mostly or completely irregular punctation of the elytra is generally a rare feature in Neotropical Cassidini present only in a few taxa (e.g. *Metriona elatior* (Klug, 1829) or *Scaeocassis turbulenta* (Boheman, 1862)).

Externally, *Chapadacassis* subgen. n. is reminiscent of *Philaspis* because of the body shape, but *Philaspis* species have moderately and regularly convex elytra without any impressions while *Chapadacassis* subgen. n. has strongly convex elytra with moderate scutellar impressions thus elytral profile is distinctly broken (Fig. 2). *Chapadacassis* subgen. n. also differs in having lateral sides of pronotum rounded (angulate in *Philaspis*), tarsal claws with small tooth (large in *Philaspis*), antennae with five basal shiny and slim antennomeres (six in *Philaspis*), and antennomeres II and III subequal in length and IV longer than either (III and IV subequal in length and II distinctly shorter than either one).

**Figures 1–7.**
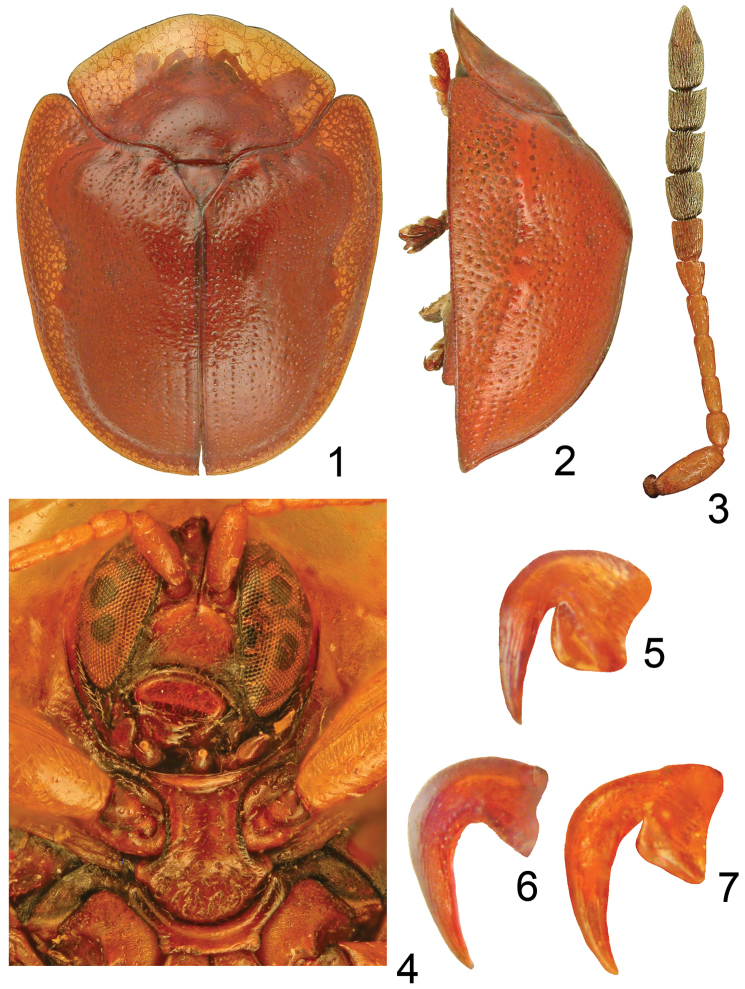
Charidotella (Chapadacassis) paradoxa sp. n. **1** body dorsal **2** body lateral **3** antenna **4** head and prosternum **5** outer claw of protarsus **6** inner claw of mesotarsus **7** inner claw of metatarsus.

**Figures 8–17.**
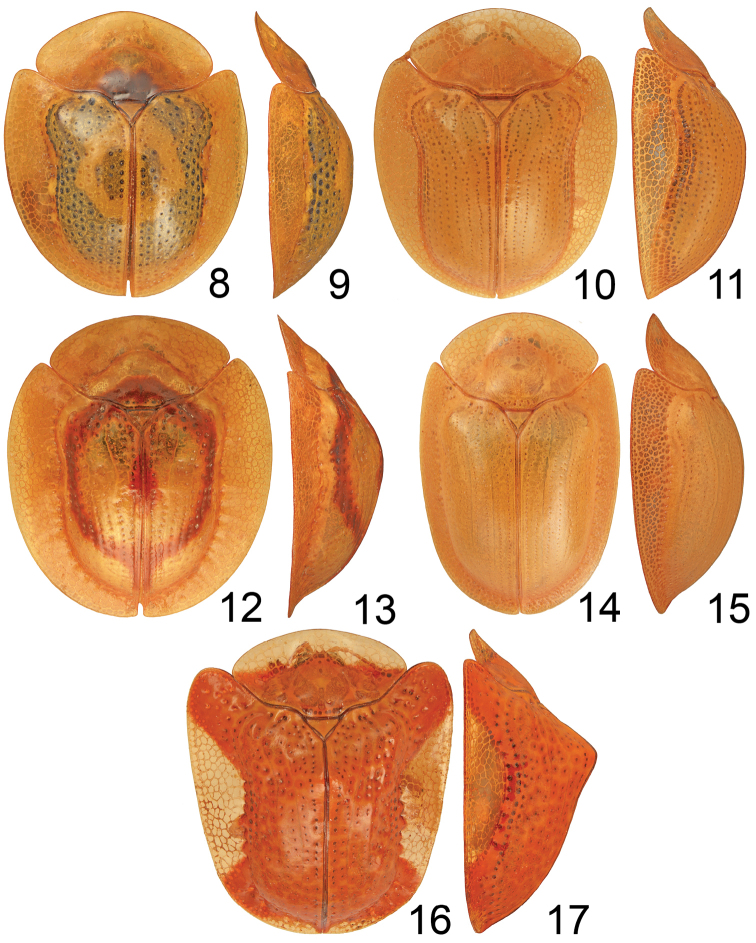
Type species for subgenera of *Charidotella*. **8–9** Charidotella (s. str.) zona (Fabricius, 1801) **10–11** Charidotella (Chaerocassis) marculenta (Boheman, 1855) **12–13** Charidotella (Xenocassis) amoena (Boheman, 1855) **14–15** Charidotella (Philaspis) seriatopunctata (Spaeth, 1901) **16–17** Charidotella (Metrionaspis) rubicunda (Guérin-Méneville, 1844).

#### Description.

Body 7.6 mm long and 5.9 mm wide, broadly oval and strongly convex (Figs 1–2). Pronotum subpentagonal, 1.9 times wider than long, widest slightly before midlength with obtuse lateral sides. Disc indistinctly separated from explanate margin, whole surface of pronotum sparsely and coarsely punctate. Scutellum triangular, smooth, dull, micro-reticulate. Elytral base distinctly wider than base of pronotum, humeral angles strongly protruding anterad and rounded. Disc strongly convex, moderately impresed on each side of scutellum, thus lateral profile broken (Fig. 2). Punctation of elytra overall coarse, mostly irregular only first two rows more or less regular in apical half. Marginal row distinct, interrupted by large callosity around midlength, its punctures approximately twice coarser than those on disc. Explanate margin broad, almost as broad as half width of disc, strongly declivous, sparsely and coarsely punctate. Extreme outer margin swollen.

Eyes large, gena not visible. Clypeus transverse, impunctate and shiny, anterior margin micro-rugose and slightly elevated (Fig. 4). Antennae slim, antennomeres I–V slim, glabrous and shiny, antennomere V intermediate, VI–XI broad and densely pubescent (Fig. 3). Labrum oval, not emarginate. Mandible with three large teeth. Prosternal collar slightly expanded towards mouth. Prosternal process broad with moderately expanded apex. Metepisterna coarsely punctate and dull. Mesepimera and mesepisterna micro-reticulate and dull. Metaventrite smooth, shiny and sparsely punctate. Abdominal ventrites I–IV smooth and shiny, V shiny and sparsely punctate, each puncture with long seta. Legs normal, slim, tarsal claws divergent. Both pro- and metaclaws appendiculate with small tooth. Outer mesoclaw claw simple (Fig. 6), inner with small tooth.

### Charidotella (Chapadacassis) paradoxa sp. n.

Taxon classificationAnimaliaColeopteraChrysomelidae

http://zoobank.org/2F581A64-5A62-4D6C-BDBA-0570F65417EA

#### Type locality.

The type locality most likely refers to Chapada dos Guimarães (approximately 15°10'–15°30'S, 55°40'–56°00'W), Mato Grosso, Brazil.

#### Type material.

Holotype, pinned: “BRAZIL, Mato Grosso | Chapada Plateau | XI 1965 | native collector [white, printed and cardboard label]” (preserved at Department of Biodiversity and Evolutionary Taxonomy, Wrocław, Poland). Paratype, pinned: same data as holotype (preserved in collection of L. Sekerka, Prague, Czech Republic). Both specimens are provided with an additional red, printed and cardboard label: “HOLOTYPUS [or PARATYPUS respectively] | Charidotella | Chapadacassis sgen. n. | paradoxa sp. n. | L. Sekerka & | L. Borowiec des. 2014”.

#### Description.

Body 7.6 × 5.9 mm, broadly oval and strongly convex (Figs 1–2).

Dorsum uniformly reddish-yellow. Margins of thoracic segments, trochanters, head, central parts of abdominal ventrites, and tarsi infuscate. Remaining ventral parts yellow. Five terminal antennomeres black, remaining yellow.

Pronotum subpentagonal, 1.9 times wider than long, widest slightly before midlength with obtuse lateral sides. Disc indistinctly separated from explanate margin, strongly convex, without impressions, sparsely and coarsely punctate, punctures laterobasally gradually coarser. Interspaces smooth and shiny, 1–4 times wider than puncture diameter. Explanate margin broad, lateral sides coarsely and sparsely punctate, transparent, smooth, and shiny, and with honeycomb structure. Anterior margin regularly convex.

Scutellum triangular, smooth, dull, micro-reticulate.

Elytra widest in basal third, then slowly tapering posteriorly. Elytral base distinctly broader than base of pronotum, humeral angles strongly protruding anterad and rounded. Disc strongly convex, with moderate impression on each side of scutellum, thus profile broken in lateral view (Fig. 2). Punctation of elytra overall coarse, mostly irregular only first two rows more or less regular in apical half. Punctures gradually coarser from top of disc to lateral sides. Interspaces 1–5 times wider than puncture diameter, finely micro-reticulate and appear shiny. Marginal row distinct, interrupted by large callosity around midlength, its punctures approximately twice coarser than those on disc (Fig. 2). Explanate margin broad, almost as broad as half width of disc, strongly declivous, sparsely and coarsely punctate, punctures gradually denser towards base and apex. Interspaces 1–5 times wider than puncture diameter, micro-reticulate and appear dull. Extreme outer margin swollen.

Clypeus 1.3 times broader than long, impunctate and shiny, anterior margin micro-rugose and slightly elevated. Antennae slim, length ratio of antennomeres: 100:46:49:59:54:45:57:57:55:56:115. Antennomere III slightly longer than II, VII–X subequal in length and approximately as long as wide (Fig. 3). Labrum oval, its lower margin smooth, not emarginate. Prosternal collar slightly expanded towards mouth. Prosternal process broad with moderately expanded apex, its surface microreticulate, sparsely and coarsely punctate, each puncture with single long seta (Fig. 4).

Legs normal, slim, tarsal claws divergent. Both fore claws appendiculate (Fig. 5). Inner mid claw simple (Fig. 6), outer with small tooth. Inner hind claw with large tooth (Fig. 7), outer with small.

#### Diagnosis.

At first glance Charidotella (Chapadacassis) paradoxa sp. n. reminds some species of the subgenus *Philaspis*. Particularly recently described, Charidotella (Philaspis) marginepunctata Borowiec, 2004 (also from Chapada in Mato Grosso) because of quite similar body shape and coarsely punctate explanate margin of elytra and pronotum. The latter distinctly differs in regularly punctate and less convex elytra without postscutellar impressions, subhorizontal explanate margin of the elytra, and the presence of a small black spot on each elytron.

#### Etymology.

The species epithet from Latin “*paradoxus*” = peculiar or curious for its unusual combination of morphological characters for Neotropical Cassidini.

#### Distribution.

Brazil (Mato Grosso).

### Key to subgenera of *Charidotella* Weise, 1896

**Table:** 

1	Eyes large covering whole sides of the head, gena very narrow or invisible	**2**
–	Eyes moderately sized, covering 2/3 of lateral sides of the head, gena well visible, covering the basal third	***Xenocassis* Spaeth, 1936**
2	Punctation of elytra regular	**3**
–	Punctation of elytra completely irregular	***Chapadacassis* subgen. n.**
3	Body outline subcircular to subtriangular. Pronotal sides usually broadly rounded	**4**
–	Body outline oval, parallel-sided. Pronotal sides sub-angulate	***Philaspis* Spaeth, 1913**
4	At least in male one of the metaclaws simple	**5**
–	All claws in both sexes with basal tooth or in male external claw of mesotarsi simple	***Charidotella* s. str.**
5	Elytra regularly convex or slightly impressed around scutellum. Antennae with six shiny basal antennomeres and five dull and broad apical	***Chaerocassis* Spaeth in Hincks, 1952**
–	Elytra with a large postscutellar gibbosity, thus lateral profile appears angulate. Antennae with five shiny basal antennomeres and six dull and broad apical	***Metrionaspis* Spaeth, 1942**

## Supplementary Material

XML Treatment for Charidotella (s. str.)

XML Treatment for Chaerocassis

XML Treatment for Metrionaspis

XML Treatment for Philaspis

XML Treatment for Xenocassis

XML Treatment for Chapadacassis

XML Treatment for Charidotella (Chapadacassis) paradoxa
